# Intrinsic Performances of Reverse Osmosis and Nanofiltration Membranes for the Recovery and Concentration of Multicomponent Mixtures of Volatile Fatty Acids: A Semi-Pilot Study

**DOI:** 10.3390/membranes15080221

**Published:** 2025-07-23

**Authors:** Omar Atiq, Gonzalo Agustin Martinez, Lorenzo Bertin, Serena Bandini

**Affiliations:** 1 Department of Civil, Chemical, Environmental and Materials Engineering—DICAM, Alma Mater Studiorum University of Bologna, Via Terracini 28, 40131 Bologna, Italy; gonzalo.martinez3@unibo.it; 2 Department of Food and Pharmaceutical Sciences, University of Parma, Parco Area delle Scienze 27/A, 43124 Parma, Italy; lorenzo.bertin@unibo.it

**Keywords:** concentration polarization, real rejection, membrane permeability, spiral-wound modules, ionic equilibria, water recovery, fermentation downstream

## Abstract

This study presents data from Reverse Osmosis (RO) and Nanofiltration (NF) spiral-wound polyamide modules tested in a semi-pilot plant with multicomponent mixtures of Volatile Fatty Acids (VFAs) comprising acetic, propionic, butyric, valeric, and hexanoic acids. A robust method combining film theory and dissociation equilibria was developed to estimate interfacial concentrations, enabling accurate analysis of concentration polarization, real rejection, and effective transmembrane driving force. Concentration polarization strongly affects NF membranes, resulting in real rejections up to 20% higher than apparent values, while its effect is negligible for RO membranes. NF rejections show marked sensitivity to pH and VFA feed concentration: at 20 g/L and highest flux, acetic acid real rejection increases from 80% to 91% as pH rises from 6 to 9. At pH 7, rejections decline with feed concentration, with acetic acid dropping from 55% at 20 g/L to 32% at 63 g/L, at the same flux. These changes correlate with the molecular weight of the acids. Conversely, RO rejections are marginally affected by pH and not influenced by concentration due to dominant steric exclusion. Membrane permeabilities remain unaffected by VFAs and align with pure water values. The data analysis framework is effective and applicable across a wide range of conditions and membranes.

## 1. Introduction

Volatile Fatty Acids (VFAs) serve as essential intermediates in the synthesis of biopolymers, reduced chemicals, and biofuels. Their production from agro-industrial side-streams and biological wastes, such as manure, offers a sustainable approach that promotes the advancement of a circular bioeconomy [1,2,3,4,5,6,7,8]. Despite mixed-culture anaerobic fermentation being a sustainable and robust method, the relatively low concentrations achievable—typically below 10 g/L and rarely exceeding 20–40 g/L, even under optimal conditions—have limited its commercial viability. Given the market demand for VFA concentrations exceeding 100 g/L, there is an urgent need to develop processes capable of efficiently recovering and concentrating VFA-rich streams from anaerobic fermenters, which must be carefully optimized to meet the specific requirements of their intended downstream applications [9,10].

Several separation and concentration technologies have been explored to this end, including liquid–liquid extraction, adsorption, electrodialysis, and forward osmosis [11,12,13,14,15]. Among these, pressure-driven membrane processes—specifically reverse osmosis (RO) and nanofiltration (NF)—have gained particular attention and have been widely investigated for the concentration and/or fractionation of VFA mixtures [14,16,17,18,19,20,21,22,23,24,25] as they emerge as particularly promising due to their energy efficiency, modularity, and ability to selectively retain VFAs. Overall, the literature shows that both techniques are effective for concentrating weak organic acids at high pH values. RO membranes typically exhibit lower permeation rates compared to NF; nevertheless, it effectively removes VFAs from fermentation broths while simultaneously producing high-purity demineralized water that can be recycled within the production process, thereby enhancing overall efficiency. NF operates through more complex mechanisms when treating multicomponent mixtures of organic acids. Its performance is strongly influenced by factors such as pH—which affects acid speciation and contributes to the formation of the fixed charge of the membrane—as well as the total solute concentration and the presence of multivalent ions, which induce additional non-specific adsorption phenomena related to membrane charge formation [24,26,27]. In this framework, recent studies have thoroughly reported on the influence of total concentration and the relative proportions of acids on the performance of both RO and NF membranes. Specifically, it has been observed that, for NF, the rejection of individual acids in mixture decreases as the total concentration rises, while the relative proportions of the acids have little to no impact—at least in ternary systems such as acetic–propionic–butyric and acetic–butyric–lactic [19,20,21]. This behavior was further confirmed by Wu et al. [23], who reported a 62% rejection decrease of the acetic acid when the concentration varied from 9 to 80 g/L. In contrast, no effect of concentration was detected with RO membranes.

Although the mentioned studies have provided extensive datasets using various membranes within the same category, they mostly relied on small-scale laboratory setups, often operated in dead-end mode. Notably, Domingos et al. [25] carried out experiments on commercial RO and NF spiral-wound modules under tangential filtration conditions, systematically examining the effects of operating parameters—pH, temperature, transmembrane pressure, and concentration—on the simultaneous fractionation and concentration of synthetic multicomponent VFA mixtures. Moreover, all these studies—including that of Domingos et al. [25]—reported membrane performance exclusively in terms of observed rejection values and total volume fluxes, which are often influenced by concentration polarization phenomenon, resulting in misleading interpretations of the intrinsic behaviour of the membrane.

For large-scale process development and scale-up across diverse operational scenarios, process engineers need predictive models of membrane modules, independent of geometry or size. The development of such models relies on the dependence of real rejection data—unbiased by concentration polarization effects—on operating conditions, and requires information on acid dissociation equilibria and the impact on membrane permeability. Therefore, there is a critical need for comprehensive experimental data—ideally derived from industrial-scale modules—coupled with the development of rigorous criteria for data analysis and interpretation, to precisely evaluate intrinsic membrane performance. Aiming to fill this gap, the objectives of this work were the following:

- To provide additional experimental data to complement the existing dataset published by our research group on spiral-wound polyamide RO and NF modules treating multicomponent VFA mixtures—including acetic, propionic, butyric, valeric, and hexanoic acids—under semi-pilot batch plant conditions [25]. These data include both the individual compositions of the permeate stream exiting the membrane module and the cumulative compositions of the permeate collected throughout the concentration process, against the corresponding feed concentrations. The former are essential for evaluating membrane performance, while the latter are crucial for assessing the overall concentration process efficiency.
- To propose a methodology and apply it to the experimental results presented herein, as well as to relevant additional data previously published [25], aimed at calculating the real rejection of each species under varying operating conditions by determining the extent of concentration polarization, and assessing whether and how the presence of organic acids influences membrane permeability and the intrinsic separation performance.

## 2. Materials and Methods

The quantification of the intrinsic membrane performances relies on experimental data obtained in a semi-pilot plant. The experimental procedure has been previously described in detail by our research group [25], where only the constant-concentration data were extensively reported. For the sake of clarity, the key information necessary to understand and reproduce the experimental work documented in this study is summarized below.

All experiments were performed starting with artificial solutions containing acetic (AA), propionic (PA), butyric (BA), valeric (VA), and hexanoic (HA) acids at equal mass proportions; the physical–chemical properties of the acids are reported in Table S1 [28,29,30,31] of the Supplementary Materials. The artificial solutions were prepared to mimic the typical conditions of a stream produced by anaerobic digestion of organic matrices, such as grape pomaces, vegetable or urban wastes [8]—specifically, at total concentration of 20 g/L with the pH ranging from 6 to 7. Reagent-grade pure compounds were dissolved in demineralized water (conductivity <80 µS/cm) and the pH was adjusted to the desired value by NaOH addition.

Spiral-wound modules (12 inches in length and 1.85 inches in diameter, 0.38 m^2^ nominal membrane area, 34 mil feed spacer), manufactured by Veolia Water Technologies SpA (Milan, Italy) were used; the corresponding values of the feed spacer porosity and the hydraulic diameter were calculated as 86.9% and 0.985 mm, respectively [32]. Modules were arranged with standard AG and DK membranes, thin-film composite membranes with a polyamide active layer supported on polysulfone. Standard AG membranes are brackish-water reverse-osmosis membranes, whereas DK are industrial high-rejection nanofiltration membranes, which typically offer 150–300 Da molecular weight cut-off. The main characteristics of the membranes, such as nominal rejections, Isoelectric Point (IEP) and pure water (hydraulic) permeabilities are reported in Table S2 [25,32,33,34,35,36,37,38] of the Supplementary Materials.

**Figure 1:**
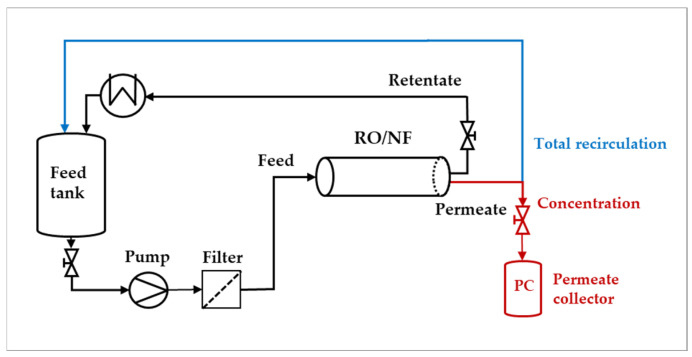
Scheme of the semi-pilot plant used for “total recirculation” and “concentration” mode trials.

The semi-pilot plant employed is depicted in Figure 1, and has been described in detail in [25,34]. The main elements are a 50 L tank for the feed solution, a positive displacement pump, a guard filter (2.5 µm) to protect the module, a heat exchanger to control the temperature in the feed tank, a permeate collector, a needle valve on the retentate to regulate the pressure to the module, and typical instruments for the measure of flow rates to the pump and in the permeate stream. In the “total recirculation mode”, both the retentate and the permeate streams were recirculated to the feed tank to maintain the total concentration at a constant value, whereas in the “concentration mode”, the permeate was collected down-stream from the module, to perform experiments at increasing concentration in the feed tank.

Given that the module recoveries in the experiments were relatively low (typically less than 5%), the spiral-wound module has been described using lumped parameters; namely, the module performances referred to the properties (composition, flow rate and temperature) existing in the feed stream (Feed—Figure 1). In addition, as the total volume of pipes + membrane module + utilities and instruments was much lower than the volume of the feed tank, at any time, the properties of the “feed stream” are considered as matching those of the feed tank.

All experiments were performed at 400 L/h feed flow rate, corresponding to an effective velocity in the feed side of 0.237 m/s [32] while varying operating conditions—pH, temperature and feed pressure. The Trans-Membrane Pressure (TMP) across the membrane was calculated as reported in Equation (1):

(1) $$TMP=\frac{P_{Feed}+P_{Retentate}}{2}-P_{permeate}$$

where the pressure in the permeate was maintained at the atmospheric value.

The permeate volume flux ($J_{v}$) was calculated as the ratio of the volume flow rate of the permeate stream and the membrane area.

The list of experiments elaborated on in this work and the corresponding operative conditions are reported in Table 1. The data in “total recirculation” mode were taken from [25], whereas the full set of data obtained in “concentration mode” are part of this work. Owing to the superior performances experienced at high pH values, the range from 6 to 9 has been selected.

Experiments in “total recirculation mode” were carried out at constant feed concentration of 20 g/L; volume flux and the individual acid concentration in the feed tank and permeate stream were measured at regular intervals within the given TMP range. Conversely, the experiments in “concentration mode” were performed at constant TMP, starting from 20 g/L; total volume flux and the individual acid concentration in the feed tank, permeate stream and permeate collector were measured. In all cases, concentrations were analysed through GC-FID equipment, as described in [25]. The temperature of 30 °C was selected, as it reflects typical conditions of fermentation broths, while 45 °C was included to assess membrane sensitivity to thermal variations. Moreover, such elevated temperatures may be relevant in processes involving thermophilic microbial strains.

## 3. Membrane Performance: Data Processing Criteria

This section outlines the methodology used to process experimental data for evaluating membrane performance. The analysis relies on measurements of total volumetric flux, pH, and the concentrations of individual volatile fatty acids in both the feed and permeate streams. The procedure begins by calculating the real rejection for each species, followed by an analysis of the total flux data to assess whether the operating conditions affect membrane permeability. In all cases, the solutions were modelled as mixtures of organic acids that dissociate in a pH-dependent manner, with sodium ions serving as the corresponding counter-ions.

### 3.1. Real Rejections

The experimental data of the total concentration of each organic acid in the feed side ($c_{A_{i},tot}^{f}$) and in the permeate stream ($c_{A_{i},tot}^{p}$) allow the calculation of the “observed rejection”, as reported in Equation (2):

(2) $$R_{obs,A_{i}}=1-\frac{c_{A_{i},tot}^{p}}{c_{A_{i},tot}^{f}}$$

Such a value, however, seldom reflects the real membrane performance, since it can be greatly affected by the concentration polarization in the feed-side. The appropriate parameter to characterize the membrane performances is the “real rejection”, defined in Equation (3), which accounts for the total concentration of the organic acid or sodium ion present at the feed/membrane interface ($c_{i,tot}^{m}$):

(3) $$R_{real,i}=1-\frac{c_{i,tot}^{p}}{c_{i,tot}^{m}}\;\mathrm{with}\;i=A_{i}\;\mathrm{and}\;Na^{+}$$

The total concentration of each organic acid at the interface can be estimated by the film theory model:

(4) $$\frac{J_{v}}{k_{L,A_{i}}}=\ln\;\;\frac{c_{A_{i},tot}^{m}-c_{A_{i},tot}^{p}}{c_{A_{i},tot}^{f}-c_{A_{i},tot}^{p}}$$

in which $J_{v}$ is the experimental value of the total volume flux across the membrane and $k_{L,A_{i}}$ is the mass transfer coefficient of the acid in the module, which can be calculated using the correlation reported in Table S3 of the Supplementary Materials, which was originally developed for spiral-wound modules of the same type within a confidence range of 20% [32]; a correction factor is also applied to account for the difference between the bulk feed viscosity and that at the membrane interface. The mixing rules for calculating density and viscosity of the solutions are extensively reported in Table S4 [29].

For any experimental condition, each set of experimental data can be elaborated to determine the real rejection value of the individual organic acid as a function of the experimental total volume flux. Correspondingly, the real rejection of the sodium ion can be obtained. To this aim, the sodium ion concentrations in the permeate stream ($c_{Na^{+}}^{p}$) and at the feed/membrane interface ($c_{Na^{+}}^{m}$) are calculated by solving the ionic equilibria reported in Table 2. Specifically, the feed interfacial pH is assumed equal to the bulk value, due to the high mobility of protons.

The influence of concentration polarization on membrane performance is effectively characterized by the polarization coefficient (Equation (5)), $\Gamma_{A_{i}}$, defined as the ratio of the total acid concentration at the feed/membrane interface to that in the bulk feed solution:

(5) $$\Gamma_{A_{i}}=\;\frac{c_{A_{i,tot}}^{m}}{c_{A_{i,tot}}^{f}}$$

A polarization coefficient approaching unity indicates minimal polarization, reflecting a uniform distribution of the acid within the feed solution. Conversely, a value exceeding unity signifies substantial accumulation at the interface.

### 3.2. Membrane Permeability

To evaluate the potential impact of organic acids on membrane permeability, the procedure developed by Bandini and Morelli was employed [34].

Given that the observed rejections of the individual species are significantly high (Section 4), the total volume flux is described according to the solution–diffusion model (Equation (6)), namely, assuming a unitary value of the Staverman coefficient.

(6) $$J_{v}=L_{p}\cdot \Delta P_{eff}=L_{p}\cdot (TMP-\Delta \pi )$$

where $L_{p}$ represents the membrane permeability, whereas $\Delta P_{eff}$ is the effective driving force accounting for the osmotic pressure difference across the membrane, Δπ, which should be calculated at the concentration values existing at the membrane interface. In this regard, within the concentration range studied here, the Van’t Hoff equation can be applied with confidence for the VFA mixture (Equation (7)). For instance, literature data show that for acetic acid, the Van’t Hoff equation remains valid up to total concentrations of 100 g/L [39].

(7) $$\Delta \pi =R_{g}T\left[\sum_{A_{i}=1}^{5}\left(c_{A_{i,tot}}^{m}-c_{A_{i,tot}}^{p}\right)+(c_{Na^{+}}^{m}-c_{Na^{+}}^{p})\right]$$

where *R_g_* denotes the gas universal constant.

Obviously, the hydraulic permeability, $L_{pw}$, was obtained from Equation (6), elaborating pure water flux data, as reported in Equation (8).

(8) $$J_{v,w}=L_{pw}\cdot TMP$$

Under isothermal conditions and assuming the absence of pore swelling caused by pH and/or solute concentration, the membrane permeability can be related to the hydraulic permeability according to the relationship in Equation (9), derived from the porous vision described in the Steric Pore Model [34]:

(9) $$L_{p}=L_{pw}\cdot \eta_{w}/\eta^{m}$$

in which $\eta^{m}$ and $\eta^{w}$ represent the viscosities of the solution at the membrane interface and of pure water, respectively. Strictly speaking, these values should be evaluated under the conditions prevailing within the membrane pores, where confinement effects typically result in higher viscosities compared to unconfined systems [40]; however, under the assumption that such confinement effects are comparable for both pure water and the relatively dilute solutions examined in this study, the viscosity ratio can be reasonably approximated using bulk (i.e., unconfined) viscosity values.

That said, based on the interfacial concentrations calculated as described in Section 3.1, the osmotic pressure difference can be obtained from Equation (7) and subsequently introduced into Equation (6), to estimate the membrane permeability. The comparison between the resulting membrane permeability and the hydraulic permeability, accounting for Equation (9), can provide insights into the potential effect of the acids. As an example, in the case of diluted solutions—where the viscosity is very close to that of pure water—if the estimated membrane permeability closely matches the hydraulic permeability, it can be concluded that the mixture of volatile fatty acids does not significantly affect the permeability. A graphical representation of this criterion has been used in the elaborations of this work, and it will be presented in Section 4.

## 4. Results and Discussion

**Figure 2:**
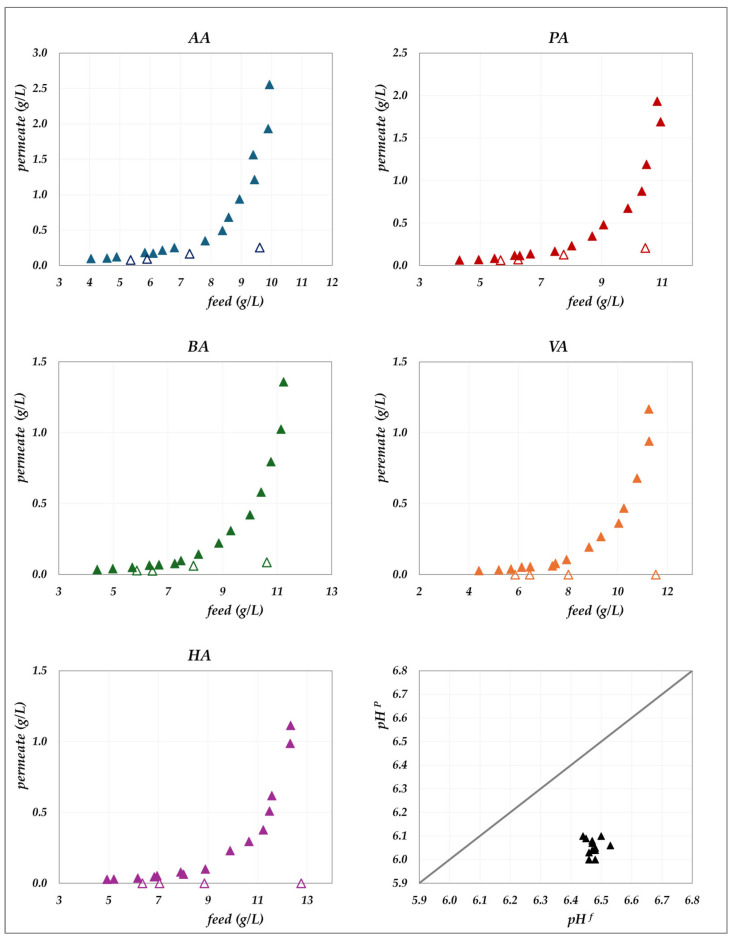
AG membrane in concentration mode at 30 °C and pH = 6.5: experimental concentration in the permeate stream (filled triangles) and in the permeate collector (empty triangles) vs. feed concentration for each acid. A parity plot illustrates the relationship between the pH of the permeate and feed streams.

**Figure 3:**
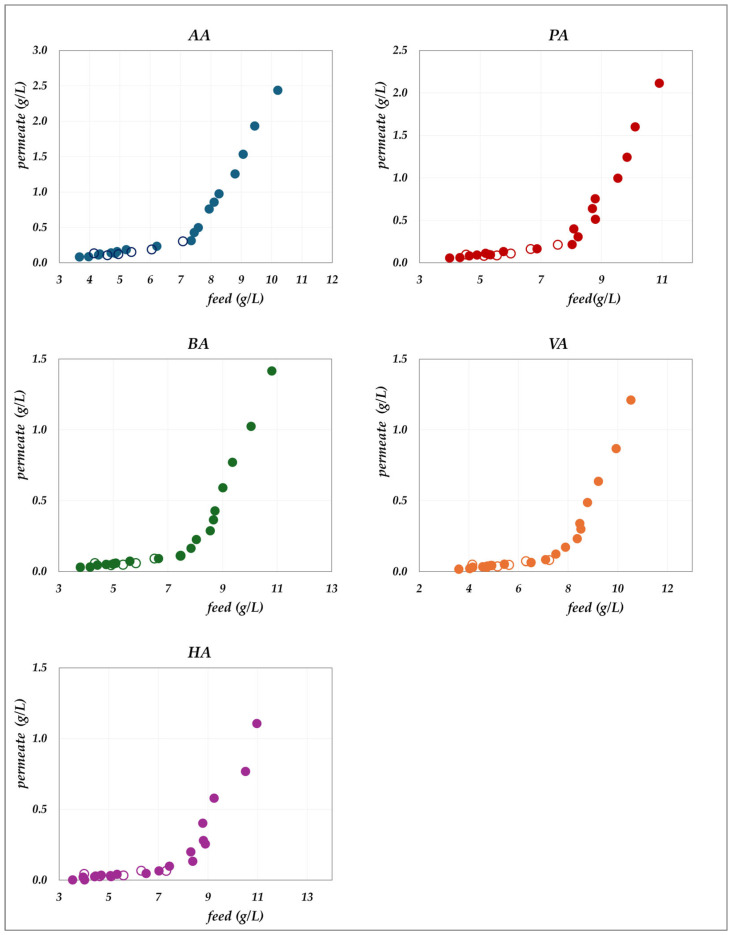
AG membrane in concentration mode at 45 °C and pH = 6.5: experimental concentration in the permeate stream (filled circles) and in the permeate collector (empty circles) vs. feed concentration for each acid.

**Figure 4:**
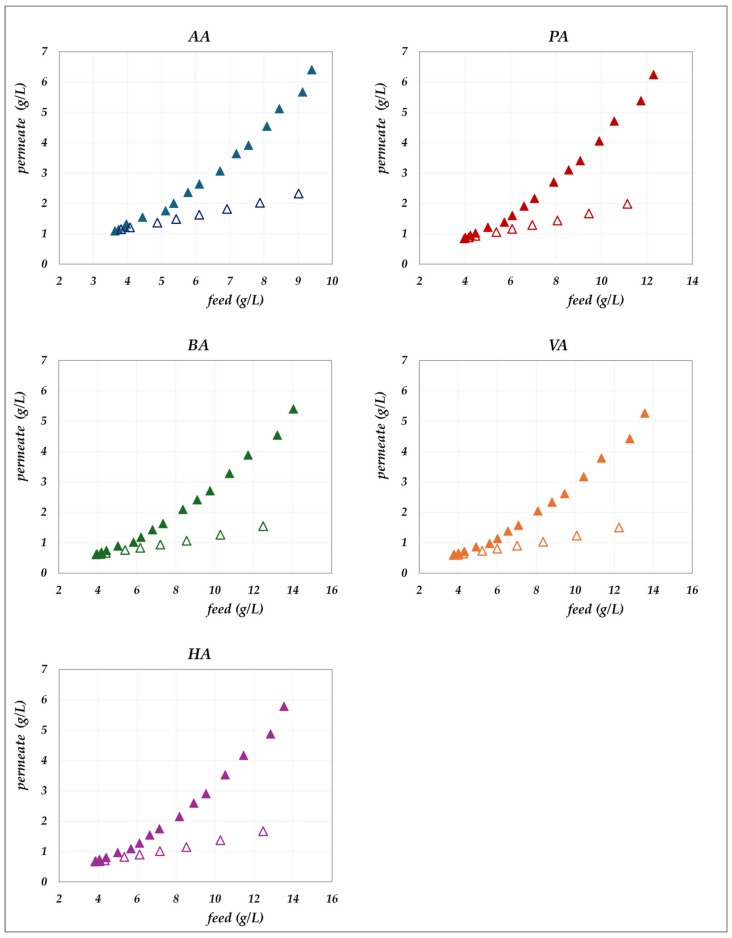
DK membrane in concentration mode at 30 °C and pH = 7: experimental concentration in the permeate stream (filled triangles) and in the permeate collector (empty triangles) vs. feed concentration for each acid.

**Figure 5:**
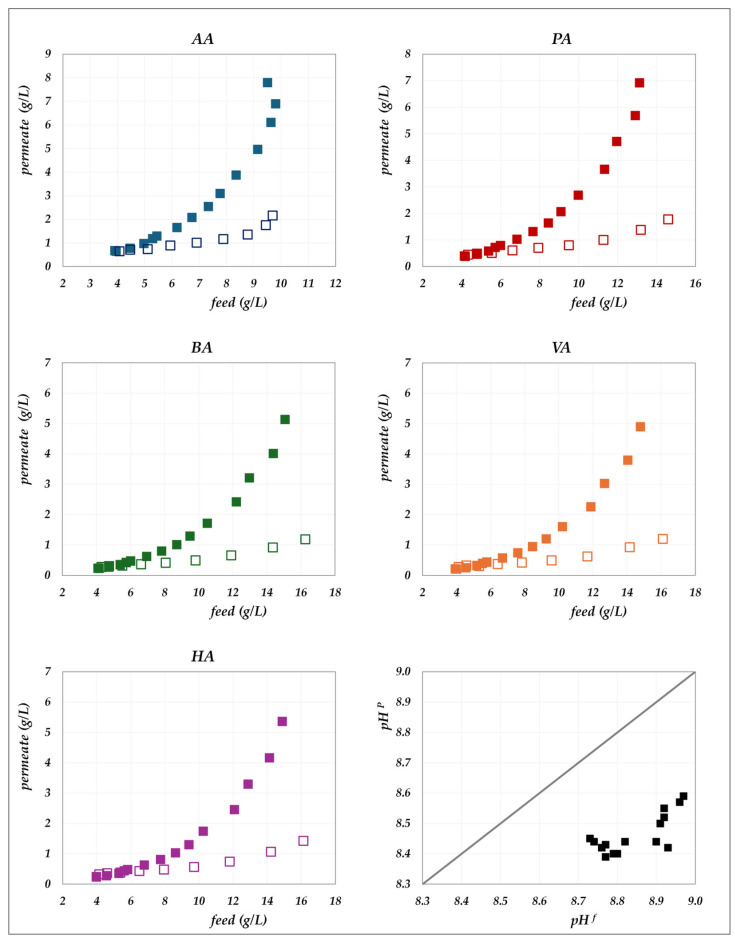
DK membrane in concentration mode at 30 °C and pH = 9: experimental concentration in the permeate stream (filled squares) and in the permeate collector (empty squares) vs. feed concentration for each acid. A parity plot illustrates the relationship between the pH of the permeate and feed streams.

The experimental data obtained in the “concentration mode” using AG–RO and DK –NF membranes are depicted in Figure 2, Figure 3, Figure 4 and Figure 5. The individual concentration of each acid in both the permeate stream and permeate collector relative to feed concentrations are shown in Figure 2 and Figure 3 for the AG membrane, and Figure 4 and Figure 5 for the DK membrane. Additionally, pH variations in the feed and permeate streams are reported. Throughout the process, the VFA concentration in the permeate collector remains lower than that of the instantaneous permeate stream. As the concentration progresses, the VFA concentration in the permeate stream increases; however, the total flux decreases significantly. Consequently, the more concentrated permeate fractions contribute progressively less to the cumulative concentration in the permeate collector. For the AG membrane, the total VFA concentration in the feed ranges from 20 to 55 g/L, corresponding to a permeate collector concentration ranging from 0.17 to 1.17 g/L. In contrast, for the DK membrane, feed concentrations vary from 20 to 65 g/L, resulting in a permeate collector concentration between 4 and 9 g/L, consistently higher than that observed for AG. Notably, the AG membrane yields high-permeate water purity. In contrast, the DK membrane allows greater passage of VFAs, resulting in lower water purity, while maintaining higher fluxes even at elevated VFA concentrations.

Clearly, the ability to simulate these trends under a variety of conditions is crucial for optimizing membrane processes. Achieving this requires precise knowledge of intrinsic membrane performance to effectively parameterize models that can guide the identification of optimal operating strategies. To this end, the following sections present real rejection results, calculated as defined in Section 3, for all experiments summarized in Table 1. It begins with the evaluation of concentration polarization extent, followed by the analysis of the influence of pH on solute rejection and membrane permeability. Finally, particular emphasis is placed on the effect of total VFA concentration, providing insights into its role in determining real rejection performance.

### 4.1. Concentration Polarization

**Figure 6:**
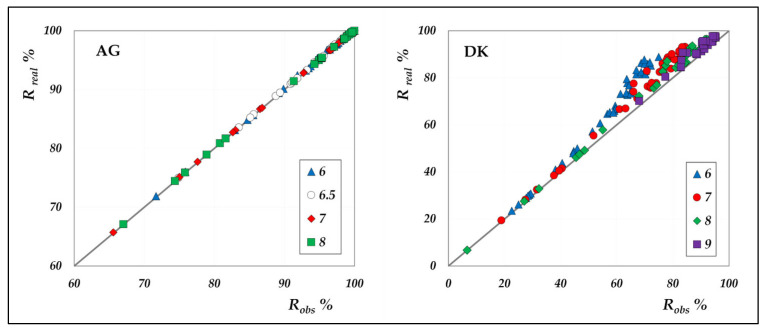
Parity plots between the real and the observed rejections of the individual species for AG and DK membranes, in the pH range from 6 to 9, at 20 g/L and 30 °C.

The comparison between real and observed rejection values for all individual species, for both AG and DK module in total recirculation mode, is shown in Figure 6.

**Figure 7:**
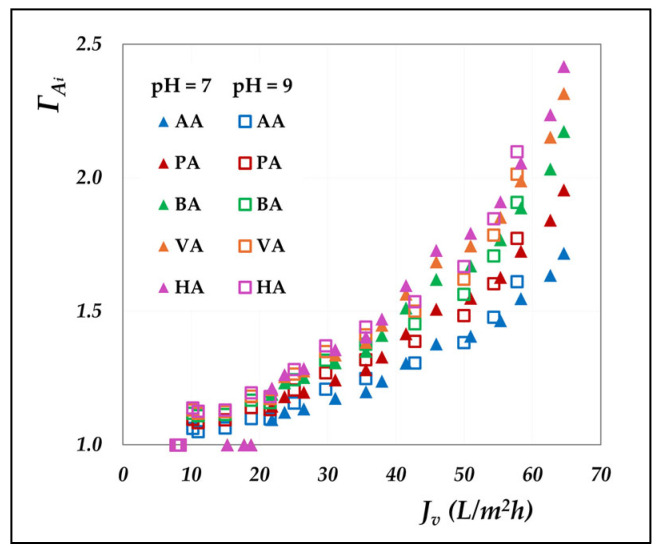
DK membrane: polarization coefficient of the individual acids vs. the experimental flux in concentration mode at 30 °C, pH = 7 and 9.

As can be clearly seen, concentration polarization is negligible in the AG membrane, whereas it significantly affects the performance of the DK membrane. Although both modules exhibit identical hydraulic diameters and superficial velocities, resulting in comparable mass transfer coefficients, the DK membrane experiences much stronger polarization effects, due to its higher transmembrane fluxes. The largest discrepancies are found at rejection levels above 60%, which correspond to high-flux conditions. To further investigate the magnitude of polarization, Figure 7 presents the polarization coefficients of the different acids at varying volumetric flux, elaborated from the concentration mode data; the cases at pH 7 and 9 are reported as examples.

Evidently, the polarization coefficients rise markedly with increasing flux, highlighting the growing influence of convective transport on concentration polarization. A direct relationship with molecular weight emerges, where heavier acids experience more pronounced polarization due to their lower diffusion coefficients (Table S1), resulting in reduced mass transfer coefficients that enhance interfacial accumulation. Moreover, the near-overlapping trends at pH 7 and 9 suggest that pH exerts minimal influence.

Overall, these findings underscore the importance of relying on real rejection data, thereby enabling accurate determination of intrinsic membrane performance. Neglecting concentration polarization can lead to significant inaccuracies and misleading conclusions regarding the behaviour of DK membranes. Consequently, studies that overlook such effects, including those reported in [19,20,21,25], should be regarded as indicative only.

### 4.2. Effect of the pH

#### 4.2.1. Effect of the pH on the Real Rejection

**Figure 8:**
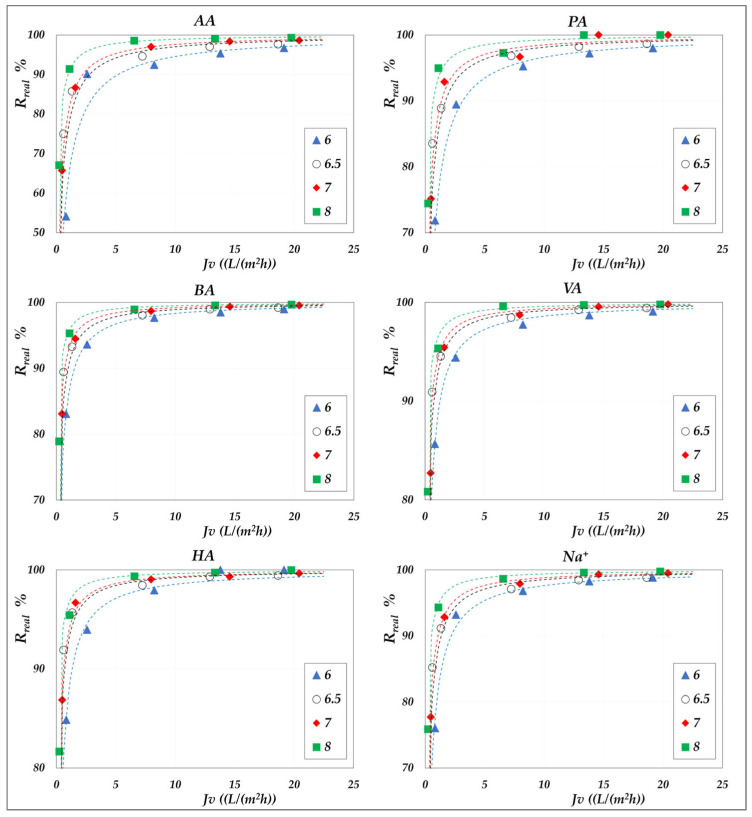
AG membrane. Real rejections vs. total volume fluxes at 30 °C, 20 g/L, pH from 6 to 8.

**Figure 9:**
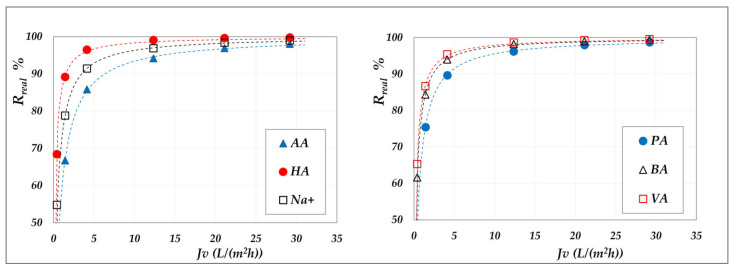
AG membrane. Real rejections vs. total volume fluxes at 45 °C, 20 g/L, pH = 6.5.

**Figure 10:**
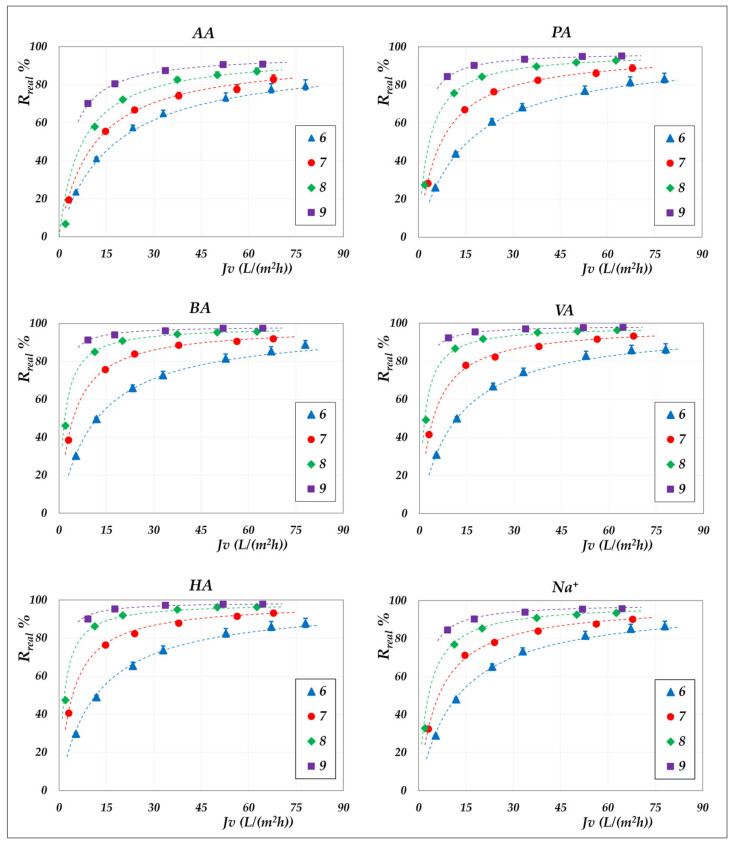
DK membrane. Real rejections vs. total volume fluxes at 30 °C, 20 g/L, pH from 6 to 9.

The effect of pH on real rejection at constant concentration—in “total recirculation” mode—is presented in detail in Figure 8 and Figure 9 for the AG membrane, and in Figure 10 for the DK membrane. Results are reported as relative to the experimental flux; dashed lines are included only to guide the data visualization. Since concentration polarization was found to be significant for the DK membrane, the mass transfer coefficients were calculated by considering the confidence interval as reported in Table S3. Accordingly, the data points refer to the values computed using average parameter values, while the error bars represent the associated uncertainty range, which remains relatively narrow.

Although the experiments were conducted at the same operating pressures for both membranes, the AG membranes clearly demonstrate greater selectivity than the DK membranes, particularly at lower fluxes. For all acids, the RO membrane achieves nearly complete rejection (approaching 100%) at higher fluxes, ensuring a high concentration efficiency of the solutes. In contrast, the DK membranes exhibit significantly higher volume fluxes—typically between 15 and 70 L·m^−2^·h^−1^—compared to the AG membranes, which operate below 10–15 L·m^−2^·h^−1^, making the NF membranes more competitive for concentration purposes, despite their lower selectivity.

Within the pH range of 6 to 8 at both 30 °C and 45 °C, the RO membrane consistently shows limiting rejections close to unity, and rather independent of pH; the lowest observed value being 0.98 for acetic acid, at pH 6.5. Temperature increase does not significantly reduce rejection, which remains stable; thus, the main benefit lies in enhanced flux. At constant pH, individual rejection values show an inverse correlation with the molar masses of the organic acids, following the order AA< PA < BA < VA < HA. Conversely, over a pH range of 6 to 9 (Figure 10), the NF membrane exhibits limiting rejections below unity, ranging from 0.90 to 0.98 and reconfirming the inverse correlation with the molar masses of the acids. At a constant concentration of 20 g/L, increasing the pH results in an enhanced rejection, suggesting that operating at pH levels above 7 is beneficial for achieving effective acid concentration and a moderately pure permeate. This trend is typically ascribed to the combined effects of an increasing net negative fixed charge of the membrane and the increasing acid speciation with the pH [41,42,43,44]. Differences in rejection performance between RO and NF at the same pH can be attributed to steric exclusion resulting from the significant disparity in average pore size, as suggested by their markedly different hydraulic permeabilities (Table S2). Finally, in accordance with the electroneutrality conditions, sodium ions consistently exhibit rejection values intermediate between those of the VFAs in all cases.

#### 4.2.2. Effect of the pH on Membrane Permeability

**Figure 11:**
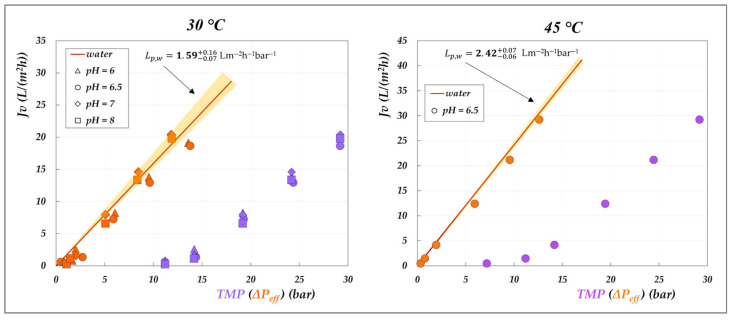
Effect of the solutions on AG membrane permeability at 30, 45 °C and 20 g/L: experimental fluxes as a function of transmembrane pressure (lilac symbols) and of the effective driving force (orange symbols, as calculated from Equations (6) and (7)). Shaded cones indicate the confidence intervals for hydraulic permeabilities (Table S2).

**Figure 12:**
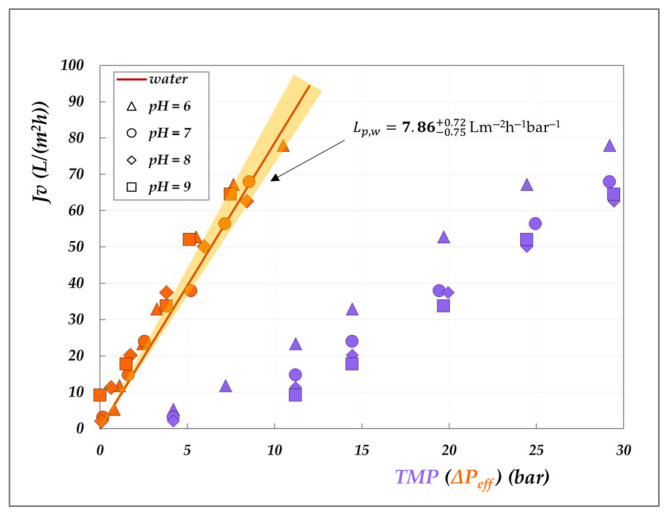
Effect of the solutions on DK membrane permeability at 30 °C and 20 g/L: experimental fluxes as a function of transmembrane pressure (lilac symbols) and of the effective driving force (orange symbols, as calculated from Equations (6) and (7). Shaded cones indicate the confidence intervals for hydraulic permeabilities (Table S2).

The graphical representation of the criterion introduced in Section 3.2 to study the role of the solutions on the membrane permeability is presented in Figure 11 and Figure 12. The analysis consists of plotting the experimental flux against the effective driving force—calculated according to Equations (6) and (7)—and comparing the resulting trend with that of pure water flux, represented by the shaded orange cones. For comparison, the corresponding experimental fluxes as a function of TMP are also reported.

Various insights can be drawn from these plots. First, the lilac data clearly highlight the significant role of osmotic pressure, evidenced by the non-zero intercept on the *x*-axis—which is particularly pronounced for the AG membrane, though still appreciable for the DK membrane. Secondly, a general inspection of the orange data points shows that most of them fall within the shaded cones, indicating that the membrane permeability ($L_{p}$) closely aligns with the hydraulic permeability ($L_{pw}$), for both membranes. Only a few isolated points exhibit a slight decrease in permeability, notably for the AG membrane at 30 °C (Figure 11), while a modest increase is observed for the DK membrane at pH 6 and 9 (Figure 12). These deviations, though very limited, cannot be attributed to viscosity effects, as the viscosity of the 20 g/L solution is essentially identical to that of pure water. In the case of the DK membrane, the observed behaviour may be attributed to limited membrane swelling. However, for both membranes, the deviations are not systematic, and we are inclined to conclude that the effect of organic acids on membrane permeability is minimal. Further dedicated investigations are required to fully elucidate this behaviour. As a final remark, it is important to emphasize that this analysis was made possible only after determining the actual osmotic pressure difference across the membrane (Δπ—Equation (7)), which depends on the accurate evaluation of the interfacial concentrations of all species involved.

### 4.3. The Role of Total VFA Concentration

**Figure 13:**
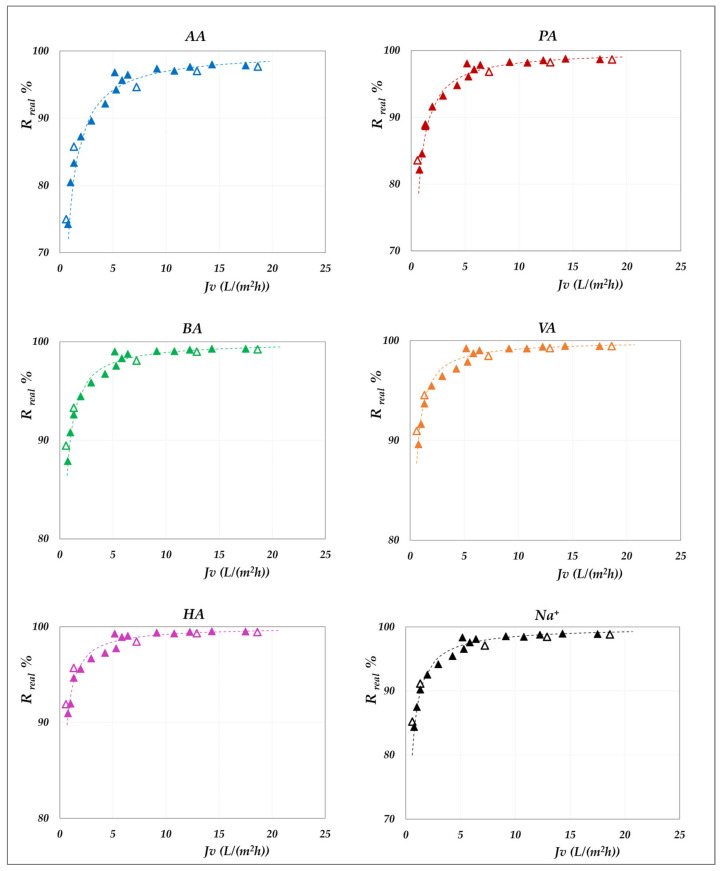
AG membrane performances, at 30 °C and pH = 6.5. Comparison between the real rejection data vs. flux at 20 g/L (“total recirculation mode”—empty triangles) and in the range from 20 to 55 g/L (“concentration mode”—filled triangles).

**Figure 14:**
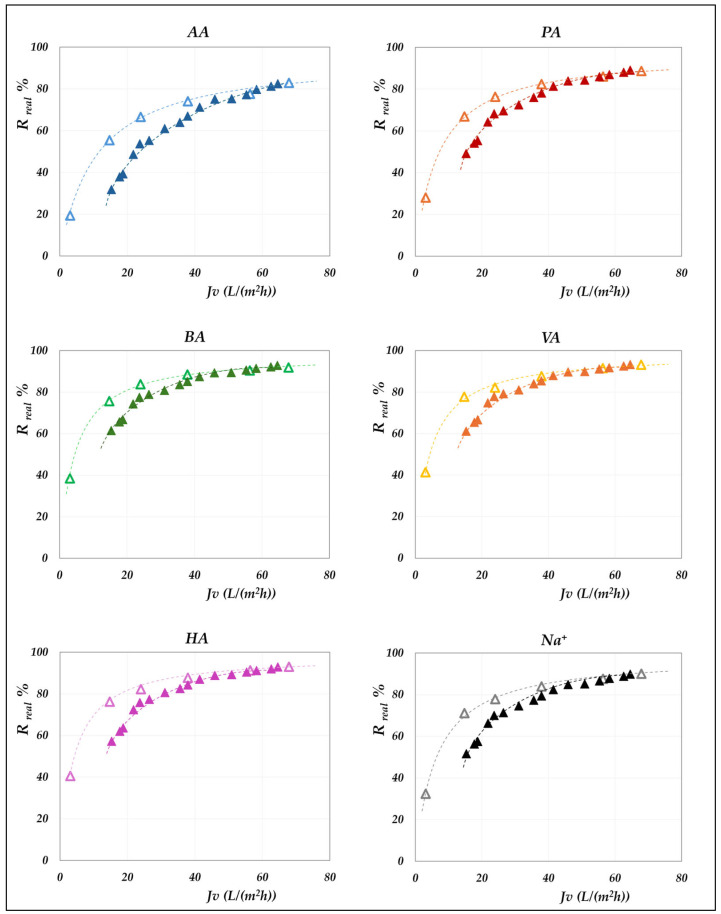
DK membrane performances, at 30 °C and pH = 7. Comparison between the real rejection data vs. flux at 20 g/L (“total recirculation mode”—empty triangles) and in the range from 20 to 65 g/L (“concentration mode”—filled triangles).

**Figure 15:**
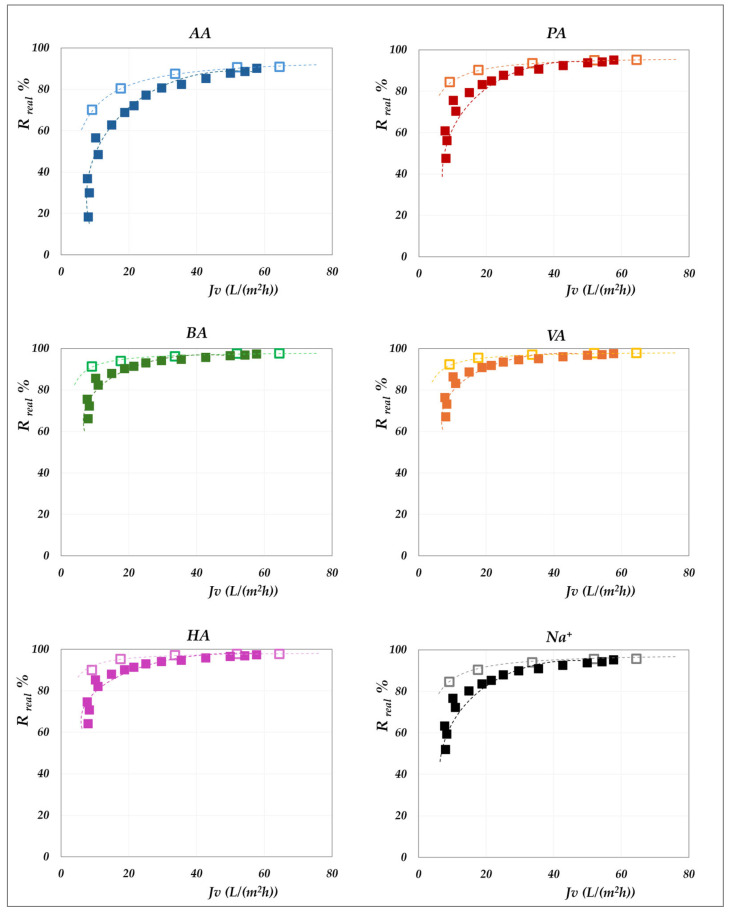
DK membrane performances, at 30 °C and pH = 9. Comparison between the real rejection data vs. flux at 20 g/L (“total recirculation mode”—empty squares) and in the range from 20 to 70 g/L (“concentration mode”—filled squares).

The influence of the total concentration of VFAs on membrane performance is examined by comparing the real rejections of the individual species measured at constant concentration (“total concentration” mode, 20 g/L) with those obtained under increasing concentration (“concentration” mode). Results for the AG membrane at 30 °C are shown in Figure 13, while Figure 14 and Figure 15 present the corresponding data for the DK membrane at 30 °C. Dashed lines have been added to improve the readability of the data trends. Regarding the filled symbols, which represent data at increasing concentrations, the flux decreases as the concentration rises.

No noticeable effect of concentration is observed for the AG membrane: rejection data at 20 g/L align precisely with those recorded across the range from 20 to 55 g/L. Similarly, data collected for the AG membrane at 45 °C closely overlap with the constant concentration results (Figure 9) and have therefore been omitted for conciseness. In contrast, the total concentration exerts a significant impact on DK membrane performance, resulting in consistent deviations from the trends observed at 20 g/L (Figure 14 and Figure 15). At a constant pH, increasing the concentration systematically reduces the real rejection of the individual species, relative to the reference concentration of 20 g/L. For instance, Figure 14 shows that at pH 7, the reduction in acetic acid rejection escalates from 10% at 32 g/L (38 Lm^−2^h^−1^) to 19% at 45 g/L (24 Lm^−2^h^−1^), reaching 42% at 63 g/L (15 Lm^−2^h^−1^); analogous trends repropose at pH 9 (Figure 15).

**Figure 16:**
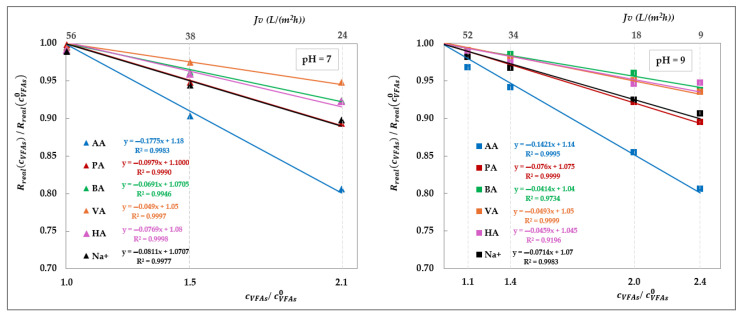
Effect of total VFA concentration on DK real rejection: rejection ratios vs. concentration ratios at pH = 7 and 9 obtained by elaborating the data from Figure 14 and Figure 15, as defined in Equation (10). Linear fittings are shown as lines.

Although previous studies have already reported a decline in observed rejection with increasing VFA concentration [19,20,21,25], the fact that an analogous trend is proposed, even with real rejections, allows us to confidently exclude concentration polarization as the underlying cause. Additionally, some of those studies showed such reduction to be independent of the relative proportions of individual acids, suggesting that the effect was not linked to specific solute identities [19,20,21]. Taken together, these findings support the conclusion that this behaviour is driven exclusively by the total concentration of VFAs on the feed side. To investigate this effect, we isolated the impact of total VFA concentration on real rejections from that of pH and total volume flux, as shown in Figure 16, which displays the rejection ratios for each solute against the corresponding concentration ratios, as defined in Equation (10):

(10) $${\left.\frac{R_{real,i}\left(c_{VFAs}^{f},\;J_{v}\right)}{R_{real,i}\left(c_{VFAs}^{f,0},\;J_{v}\right)}\right|}_{pH}vs.\;\frac{c_{VFAs}^{f}}{c_{VFAs}^{f,0}}$$

where $c_{VFAs}^{f,0}$ represents the total VFA concentration in the feed at the beginning of the concentration trial, which, in turn, matches with the concentration value used in the corresponding total recirculation trial (i.e., 20 g/L); $R_{real,i}\left(c_{VFAs}^{f,0},\;J_{v}\right)$ denotes the real rejection value obtained in the total recirculation trial at a given pH (empty symbols—Figure 14 and Figure 15) while $R_{real,i}\left(c_{VFAs}^{f},\;J_{v}\right)$ refers to the real rejection value evaluated in concentration mode at the same flux (filled symbols at matching flux in Figure 14 and Figure 15).

Figure 16 offers valuable insights, revealing that at both pH 7 and pH 9, the rejection ratios of all solutes display a strong linear correlation with the concentration ratio, following a consistent decreasing trend. Moreover, the rate of the decline depends on the solute type: acetic acid and propionic acid are the most affected, followed by butyric and valeric acids, broadly in accordance with the sequence of the molecular weights—except for hexanoic acid, which slightly deviates from this pattern. The sodium ion reconfirms an intermediate behaviour aligning with expectations based on the electroneutrality condition.

We believe that these trends support the interpretation that increasing solute concentration leads to screening of the fixed negative charges of the membrane, thereby reducing electrostatic repulsion and facilitating solute passage. This conclusion is corroborated by the less pronounced effect of the concentration obtained at pH 9 compared to pH 7, as indicated by less negative slopes of the rejection ratio trends. At higher pH, the membrane holds a more negative charge, which in turn reduces the effectiveness of charge screening at a given VFA concentration, resulting in a smaller decrease in rejection. In contrast, the absence of a concentration-dependent effect in the AG membrane (Figure 13) is expected to be attributed to dominant steric exclusion mechanisms, owing to its smaller pore size. This structural feature likely limits electrostatic interactions within the membrane matrix, thereby suppressing charge screening phenomena. As a result, the separation performance of the AG membrane appears to be governed primarily by the pH, rather than solute concentration.

## 5. Conclusions

This study presents, for the first time, a systematic evaluation of the intrinsic performance of reverse osmosis and nanofiltration membranes in concentrating multicomponent mixtures containing volatile fatty acids. A dedicated data analysis methodology was developed, incorporating mass transfer phenomena within spiral-wound geometry and thermodynamic principles from process engineering, allowing for the decoupling of concentration polarization effects from osmotic pressure in performance evaluation. The investigation utilized a comprehensive set of experimental data presented within this study, supplemented by selected results previously published by the authors.

The significant role of concentration polarization has been demonstrated in nanofiltration membranes, attributable to their inherently higher permeate fluxes compared to reverse osmosis. Under conditions of maximum permeate flux, polarization coefficients were determined to exceed approximately 1.5—from 1.7 for acetic to 2.4 for hexanoic acid.

Correlating experimental fluxes with the effective driving force yielded valuable insights into membrane permeability, which was found to remain unaffected by the presence of VFAs and to be consistent with pure water permeability. Only minimal changes, such as swelling or matrix rearrangement, occur under the tested conditions.

The availability of data in terms of intrinsic rejections enables a definitive elucidation of the key influence of total permeate flux and pH. While these effects are observed in both membrane types, they are significantly more pronounced in NF membranes. At a total VFA concentration of 20 g/L, increasing the permeate flux from 5 to 20 L/(m^2^·h) leads to relatively modest changes in rejection for RO membranes, ranging between 90% and 100%, with a slight upward trend as pH increases. In contrast, for NF membranes, an increase in flux from 10 to 75 L/(m^2^·h) results in a substantial enhancement of acetic acid rejection—from 35% to 75% at pH 6, and from 70% to 90% at pH 9. The pH-dependent effect becomes more pronounced with increasing carbon chain length, from acetic to hexanoic acid.

Moreover, the crucial role of total VFA concentration in governing the separation efficiency of NF membranes was clearly evidenced and thoroughly analysed. An increase in VFA concentration from 20 to 60 g/L at pH 7 led to a marked decrease in rejection, ranging from 43% for acetic acid to 25% for hexanoic acid. At pH 9, a similar increase in concentration (from 20 to 50 g/L) resulted in comparatively smaller rejection losses—limited to 19% and 5% for acetic and hexanoic acid, respectively.

These effects can be comprehensively explained by acid speciation, which increases dissociation at higher pH, and by the enhanced negative charge of the membrane surface, which intensifies electrostatic repulsion and reduce solute partitioning. Conversely, at higher solute concentrations, the screening of fixed-membrane charges attenuates electrostatic interactions, thereby facilitating acid transport. The impact of both pH and total VFA correlates with the molecular weight of the acids, indicating a strong contribution of steric exclusion—an effect that remains important in NF, but essentially takes over the separation in RO.

The analytical framework proposed herein proved effective and quite general. It is readily extendable to wider solute concentration ranges, pH conditions, and other different membrane types. The results obtained provide a robust basis for accurate parameterization in future modelling efforts. Such models should include different mechanisms of partitioning—including steric and electrostatic exclusion—and provide insights into the modulation of membrane charge formation under varying conditions. This approach will support the rational design of optimized membrane processes for the treatment of complex organic mixtures.

## Figures and Tables

**Table 1:** List of experiments and operative conditions.

	AG	DK
**Total Recirculation Mode** (at total VFAs = 20 g/L, from [25])		
pH	6.0, 6.5, 7.0, 8.0	6.0, 7.0, 8.0, 9.0
temperature (°C)	30 ± 0.5 , 45 ± 0.5	30 ± 0.5
TMP (bar)	11–30	4–30
**Concentration Mode** (initial VFAs = 20 g/L, [this work])		
pH	6.5	7.0, 9.0
temperature (°C)	30 ± 0.5 , 45 ± 0.5	30 ± 0.5
TMP (bar)	29.2	29.5, 29.2

**Table 2:** Ionic equilibria for the multicomponent system VFAs-Na^+^ in water.

Dissociation Equilibrium	
$10^{\left(-pK_{a,A_{i}}\right)}=\frac{c_{A_{i}^{-}}\cdot c_{H_{3}O^{+}}}{c_{A_{i}}}$	(2.a)
Molar Balances	
$c_{A_{i},tot}=c_{A_{i}^{-}}+c_{A_{i}}$	(2.b)
Electroneutrality Condition	
$\sum_{A_{i}=1}^{5}c_{A_{i}^{-}}+c_{OH^{-}}=c_{Na^{+}}+c_{H_{3}O^{+}}$	(2.c)
Water Ionic Product and pH	
$10^{-14}=c_{H_{3}O^{+}}\cdot c_{OH^{-}}$	(2.d)
$pH=-\log_{10}\left(c_{H_{3}O^{+}}\right)$	(2.e)

concentration units = mol/L.

## Data Availability

The original contributions presented in this study are included in the article and in the Supplementary Materials. Further inquiries can be directed to the corresponding authors.

## Supplementary Materials

The following supporting information can be downloaded at: https://www.mdpi.com/article/10.3390/membranes15080221/s1, Table S1: physical–chemical properties of VFAs; Table S2: Characteristics of RO and NF membranes; Table S3: Mass transfer correlation for 12x1.85-inch spiral-wound modules and Table S4: Mixing rules for the calculation of density and viscosity.
