# Supplementary material for: Intrinsic Performances of Reverse Osmosis and Nanofiltration Membranes for the Recovery and Concentration of Multicomponent Mixtures of Volatile Fatty Acids: A Semi-Pilot Study
Source: Membranes (Basel). 2025 Jul 23;15(8):221. doi: 10.3390/membranes15080221 (PMC12388675; doi:10.3390/membranes15080221)
Supplement: Supplementary file 1 [file membranes-15-00221-s001.zip › membranes-3751951-supplementary.pdf]

Table S1. Physical-chemical properties of VFAs

specie	molecular weight (kg/kmol)	$D^{\infty}_{A_i,w}$ * 10^9 (m^2/s) [28,29]		ρ^o (kg/m^3) [29]		η^o (mPa·s) [29]		pK_{a,A_i} (mol/L) [30,31]
		at 35 °C	at 45 °C	at 35 °C	at 45 °C	at 35 °C	at 45 °C	at 25 °C
w	18	-	-	998.75	993.63	0.8196	0.6115	-
AA	60.05	1.545	2.173	1036.00	1020.48	1.0375	0.8459	4.76
PA	74.08	1.293	1.818	982.17	966.99	0.9574	0.7894	4.87
BA	88.11	1.129	1.589	946.18	933.15	1.3626	1.0855	4.81
VA	102.13	1.007	1.417	927.79	915.45	1.8282	1.4041	4.82
HA	116.16	0.920	1.294	914.09	902.65	2.5547	1.9101	4.88

$D^{\infty}_{A_i,w}$ = acid diffusivity in water at infinite dilution, ρ^o,η^o=density and viscosity of the pure compound

Table S2. Characteristics of RO and NF membranes

	rejection	*solution*	*pressure/temperature*	*IEP*	*Hydraulic permeability ($Lm^{-2}h^{-1}bar^{-1}$)*
AG	99.4%	2000 ppm NaCl	15.51 bar/25 °C	3.5-4.0 [33]	$1.59^{-0.07}_{+0.16}$ (30°C); $2.83^{-0.06}_{+0.07}$ (45°C) [25,34]
DK	98%	2000 ppm $MgSO_4$	7.6 bar/25 °C	3.0-4.2 [35-37]	$\mathbf{7.86^{-0.75}_{+0.72}}$ (30 °C) [25,32,38]

The hydraulic permeabilities were calculated from literature data by averaging the values reported for other samples of the same type; noticeably, the confidence ranges are lower than 10%. The membrane charge of DK membranes has been extensively studied: electro-kinetics measurements evidenced that the point of zero charge depends on the NaCl concentration (IEP from 3.0 to 4.2), whereas it is independent of the electrolyte type. Conversely, to the best of our knowledge, no specific zeta-potential measurements are available in literature for the AG membrane; however, based on data for similar membranes the IEP can be estimated in the range from 3.5 to 4, consistent with the membrane material.

Table S3. Mass transfer correlation for 12x1.85 inches-spiral wound modules [32].

$$k_{L,A_i} = k^f_{L,A_i}\left(\frac{\eta^f}{\eta^m}\right)^{0.27} \quad \text{(S3.a)}$$

$$Sh_{A_i} = 0.0224 Re^{0.807} Sc^{1/3}_{A_i} \quad \pm 20\% \qquad 126 \le Re \le 508\,,\ Sc_{A_i} > 500 \quad \text{(S3.b)}$$

$$Sh_{A_I} = \frac{k^f_{L,A_i} d_h}{D^{\infty}_{A_i,w}}\ ;\ Re = \frac{\rho^f v_{eff} d_h}{\eta^f}\ ;\ Sc_{A_i} = \frac{\eta^f}{\rho^f D^{\infty}_{A_i,w}} \quad \text{(S3.c)}$$

$D^{\infty}_{A_i,w}$ = acid diffusivity in water at infinite dilution, ρ^f,η^f=density and viscosity of the solution,

d_h, v_{eff} =hydraulic diameter and effective velocity

Table S4. Mixing rules for the calculation of density and viscosity [29].

$\frac{1}{\rho} = \frac{\omega_{AA}}{\rho^o_{AA}} + \frac{\omega_{PA}}{\rho^o_{PA}} + \frac{\omega_{BA}}{\rho^o_{BA}} + \frac{\omega_{VA}}{\rho^o_{VA}} + \frac{\omega_{HA}}{\rho^o_{HA}} + \frac{\omega_{w}}{\rho^o_{w}}$
$ln(\eta) = x_{AA} ln(\eta^0_{AA}) + x_{PA} ln(\eta^0_{PA}) + x_{BA} ln(\eta^0_{BA}) + x_{VA} ln(\eta^0_{VA}) + x_{HA} ln(\eta^0_{HA}) + x_w ln(\eta^0_w)$
ω and x represent the mass and molar fractions, respectively

25. Domingos, J.M.B.; Martinez, G.A.; Morselli, E.; Bandini, S.; Bertin, L. Reverse Osmosis and Nanofiltration Opportunities to Concentrate Multicomponent Mixtures of Volatile Fatty Acids. *Sep. Purif. Technol.* **2022**, *290*, 120840, doi:10.1016/J.SEPPUR.2022.120840.
28. R. Wilke, C.; Change, P. Correlation of Diffusion Coefficients in Dilute Solutions. *AIChE J.* **1955**, *1*, 264–270, doi:10.1002/AIC.690010222.
29. Green, D.W.; Perry, R.H. Table 2-313, Physical and Chemical Data. In *Perry's Chemical Engineers' Handbook*; McGraw-Hill Education, 2008.
30. P. Serjeant, E.; Dempsey, B. *Ionisation Constants of Organic Acids in Aqueous Solution*; Pergamon Press: Oxford;New York, 1979;
31. Perrin, D.D.; Demsey, B.; P. Serjeant, E. *pKa Prediction for Organic Acids and Bases*; Chapman and Hall: London; New York, 1981;
32. Bandini, S.; Morelli, V. Mass Transfer in 1812 Spiral Wound Modules: Experimental Study in Dextrose-Water Nanofiltration. *Sep. Purif. Technol.* **2018**, *199*, 84–96, doi:10.1016/j.seppur.2018.01.044.
33. Fargues, C.; Sagne, C.; Szymczyk, A.; Fievet, P.; Lameloise, M.-L. Adsorption of Small Organic Solutes from Beet Distillery Condensates on Reverse-Osmosis Membranes: Consequences on the Process Performances. *J. Membr. Sci.* **2013**, *446*, 132–144, doi:10.1016/j.memsci.2013.05.051.
34. Bandini, S.; Morelli, V. Effect of Temperature, pH and Composition on Nanofiltration of Mono/Disaccharide: Experiments and Modeling Assessment. *J. Membr. Sci.* **2017**, *533*, 57–74, doi:doi.org/10.1016/j.memsci.2017.03.021.
35. Hagmeyer, G.; Gimbel, R. Modelling the Rejection of Nanofiltration Membranes Using Zeta Potential Measurements. *Sep. Purif. Technol.* **1999**, *15*, 19–30, doi:10.1016/S1383-5866(98)00050-1.
36. Oatley, D.L.; Llenas, L.; Aljohani, N.H.M.; Williams, P.M.; Martínez-Lladó, X.; Rovira, M.; de Pablo, J. Investigation of the Dielectric Properties of Nanofiltration Membranes. *Spec. Issue Nanofiltration Membr. Fundam. Appl.* **2013**, *315*, 100–106, doi:10.1016/j.desal.2012.09.013.
37. Oatley-Radcliffe, D.L.; R. Williams, S.; S. Barrow, M.; M. Williams, P. Critical Appraisal of Current Nanofiltration Modelling Strategies for Seawater Desalination and Further Insights on Dielectric Exclusion. *Desalination* **2014**, *343*, 154–161, doi:10.1016/j.desal.2013.10.001.
38. Roselli, M.; Onesti, R.; Boi, C.; Bandini, S. Recovery of Lactose from Acid Whey by Nanofiltration: An Experimental Study. *Sep. Purif. Technol.* **2025**, *353*, 128303, doi:10.1016/j.seppur.2024.128303.

.
